# The PDGF Family Is Associated with Activated Tumor Stroma and Poor Prognosis in Ovarian Cancer

**DOI:** 10.1155/2022/5940049

**Published:** 2022-09-26

**Authors:** Jiajia Li, Xiuling Zhi, Yating Sun, Mo Chen, Liangqing Yao

**Affiliations:** ^1^Department of Gynecologic Oncology, Obstetrics & Gynecology Hospital, Fudan University, Shanghai 200011, China; ^2^Department of Physiology and Pathophysiology, School of Basic Medical Sciences, Fudan University, Shanghai 200032, China

## Abstract

The initiation and progression of cancer depend on the genetic alterations inherent in cancer cells, coupled with the mutual interplay of cancer cells with the surrounding tumor stroma. The platelet-derived growth factor (PDGF) family, as a mesenchymal growth factor, was involved in tumor progression by affecting the surrounding tumor stroma in some cancer types. However, the association of the PDGF family with the ovarian cancer stroma remains elusive. In our study, we first explored the expression pattern of the PDGF family using RNA expression profiles from public databases. We found that the PDGF family was highly expressed in tumor stroma compared with the corresponding epithelial components of ovarian cancer. In particular, PDGF receptors were weakly expressed in ovarian cancer tissues compared with the respective normal tissues; even in tumor mass, PDGF receptors were predominantly expressed by tumor stroma rather than ovarian cancer cells. Importantly, functional enrichment analyses and correlation analyses revealed that the PDGF family was strongly associated with activated stromal scores in ovarian cancer, including higher stromal scores, enriched pathways related to the extracellular matrix (ECM) organization and remodeling, elevated cancer-associated fibroblasts (CAFs) infiltration, and increased tumor-associated macrophages (TAMs) infiltration, especially macrophage M2. Besides, the positive correlations of the PDGF family with CAFs infiltration and macrophage M2 infiltration were observed in other various cancer types. Of note, the PDGF family was also involved in tumor progression-related pathways, such as transforming growth factor *β* (TGF-*β*) signaling, epithelial-mesenchymal transition (EMT), angiogenesis, and phosphatidylinositol 3-kinase-Akt (PI3K-Akt) signaling. Higher expressions of PDGF receptors were also observed in ovarian cancer patients with venous or lymphatic invasion. Furthermore, we uncovered the prognostic prediction of the PDGF family in ovarian cancer and constructed a PDGF family-based risk prognosis model with a hazard ratio of 1.932 (95%confidence interval (CI) = 1.27–2.95) and *P* value < 0.01 (AUC = 0.782, 0.752 for 1 year and 2 years, respectively). Taken together, we demonstrated that ovarian cancers with high PDGF family expression biologically exhibit malignant progression behaviors as well as poor clinical survival, which is attributed to the activated tumor stroma in ovarian cancer.

## 1. Introduction

Ovarian cancer is the most aggressive and lethal gynecological malignancy at present, with a projected 5-year survival rate of less than 50% [1]. The high mortality rate of ovarian cancer is primarily ascribed to the advanced stage of disease at the time of diagnosis, widespread metastasis, high chemoresistance, and high relapse rate after initial treatment [2, 3]. The mortality remains extraordinarily high despite advancements in current treatment modalities, including surgery, chemotherapy, and new biological therapies [2, 4]. Therefore, identifying novel and alternative therapeutic regimens for ovarian cancer remains a major clinical obstacle to overcome.

It has been extensively reported that the initiation and aggressiveness of cancer are determined by the bilateral interaction of cancer cells with the favorable tumor microenvironment (ME), mainly conferred by the tumor stroma [5]. Tumor stroma is composed of cellular components, such as cancer-associated fibroblasts (CAFs), immune cells, endothelial cells, and noncellular components, including extracellular matrix (ECM), cytokines, chemokines, growth factors, exosomes, and metabolites [5, 6]. As mentioned in the literature [7, 8], tumor stroma is perceived as a major contributor to a series of tumor progression-related phenotypes, such as tumor growth, metastasis, chemoresistance, and recurrence in various cancer types. Resembling most solid tumor types, ovarian cancer patients with high amounts of tumor stroma exhibited great aggressiveness and poorer clinical prognoses, suggesting that tumor stroma sustained the malignant behavior of ovarian cancer [9–11].

The platelet-derived growth factor (PDGF) family, known as a mesenchymal growth factor, consists of four ligands (PDGFA, PDGFB, PDGFC, and PDGFD) and two PDGF receptor isoforms (PDGF receptor *α* (PDGFRA) and *β* (PDGFRB)) [12]. It is well documented that aberrant PDGF/PDGFR signaling contributes to many human diseases, including cancer [13]. In general, the PDGF family mainly exerts tumor-promoting effects in two ways: direct autocrine stimulation of cancer cells and an indirect effect through activation of the tumor stroma [14]. However, autocrine stimulation is only present in a subset of cancer types such as glioblastoma and sarcoma [14]. In most solid tumors, PDGF receptors are commonly expressed by mesenchymal cells, and PDGF ligands primarily promote the proliferation, survival, and migration of mesenchymal cells rather than tumor cells [8]. Furthermore, recent studies have shown that the expression of PDGF ligands promotes vascularization and the establishment of prominent stroma in transplant models of melanoma, breast cancer, fibrosarcoma, and lung cancer, thereby promoting tumor growth and progression [15, 16]. Regardless of the above critical observations, there has been a lack of systematic investigations on the expression of the PDGF family and its association with tumor stroma in ovarian cancer.

A vast array of publicly available sequencing projects profile various cancer specimens and provide an extensive data resource for cancer-related studies [17, 18]. Here, via multiple bioinformatic methods, we explored the expression pattern of the PDGF family in ovarian cancer and its relationship with tumor stroma-related properties, such as ECM organization and remodeling, CAFs infiltration, and tumor-infiltrating immune cell abundance. In addition, we also evaluated the vital biological traits suggestive of cancer progression. Furthermore, we constructed a PDGF family-based risk prognosis model that significantly predicted the survival rates of patients with ovarian cancer.

## 2. Materials and Methods

### 2.1. Expression of the PDGF Family in Ovarian Cancer

The individual gene mRNA levels of the PDGF family in 88 normal ovarian tissues of the Genotype Tissue Expression (GTEx) dataset and 426 ovarian cancer samples from The Cancer Genome Atlas (TCGA, http://cancergenome.nih.gov/) database were obtained from the Gene Expression Profiling Interactive Analysis (GEPIA, http://gepia.cancer-pku.cn/index.html). The expression data of PDGFRA or PDGFRB and corresponding clinical information including venous invasion and lymphatic invasion were obtained from the TCGA database. The significant difference was compared by the Wilcoxon rank-sum test. *P* < 0.05 was considered statistically significant.

The protein expression levels of the PDGF family members in 25 normal ovarian tissues and 100 ovarian cancer tissues from the Clinical Proteomic Tumor Analysis Consortium (CPTAC) dataset were analyzed from the University of ALabama at Birmingham Cancer Data Analysis Portal (UALCAN, http://ualcan.path.uab.edu/analysis-prot.html). The protein expression levels of PDGFRA and PDGFRB in pancancer were also derived from the UALCAN portal. The significant difference was evaluated by the Wilcox test. *P* < 0.05 was considered statistically significant.

Immunohistochemistry (IHC) staining of the PDGF family in normal ovarian tissues and ovarian cancer was obtained from the Human Protein Atlas (HPA, https://www.proteinatlas.org/) database.

### 2.2. Receiver-Operating Characteristic (ROC) Analysis of the PDGF Family in Ovarian Cancer

The predictive power of the PDGF family genes at separating ovarian cancer from normal ovarian samples was assessed by the receiver-operating characteristic (ROC) analysis and determination of the area under curve (AUC). A value of AUC higher than 0.5 represents a better prediction, and an AUC value of 1.0 indicates a perfect predictive power. The predictive ability was considered excellent for AUC values between 0.9 and 1.0, certain for AUC values between 0.7 and 0.9, and low when AUC values between 0.5 and 0.7.

### 2.3. Gene Ontology (GO) and Kyoto Encyclopedia of Genes and Genomes (KEGG) Analyses of the PDGF Family in Ovarian Cancer

A total of 376 ovarian cancer expression profiles were obtained from the TCGA database. Based on the individual expression level of the PDGF family genes, we divided the TCGA ovarian cancer samples into the high-expression group (top 25%) and the low-expression group (top 25%). Differentially expressed genes (DEGs) between the two groups were obtained using the limma package of R software (version: 3.40.2) according to *P* value < 0.05 and |log2 Fold Change| > 1. Gene Ontology (GO) is a widely used tool to annotate functional genes, especially molecular functions, biological pathways, and cellular components. Kyoto Encyclopedia of Genes and Genomes (KEGG) Enrichment Analysis is a database resource that links genomic information with gene function. To better understand the carcinogenic effect of the target gene, GO and KEGG analyses of DEGs were conducted using the ClusterProfiler package in R software (version: 3.18.0). Pathways with *P* < 0.05 were considered significant.

### 2.4. The Correlation between PDGF Family Expression and Pathway Score

The correlation between the PDGF family expression and pathway score in 376 ovarian cancer samples of the TCGA database was conducted using the R software GSVA package and chose parameters as method = ssgsea. After the collection of pathway-related gene sets (doi:10.3390/cancers1207178), the enrichment score of each sample in each pathway was calculated in turn according to the ssgsea algorithm. The relationship between the gene and the pathway was calculated by the Spearman's correlation. Our methods were implemented using R version 4.0.3. *P* value < 0.05 was considered statistically significant.

### 2.5. The Association between PDGF Family Expression and Microenvironmental Variables

The mRNA expression profiles of 376 ovarian cancer samples were obtained from the TCGA database. For each PDGF family member, we merely chose the top 25% (*n* = 94) with high expression and the top 25% (*n* = 94) with low expression for further study. We used the Xcell algorithm to evaluate the correlation between the PDGF family expression and ME variables, including tumor-infiltrating immune cell abundance, immune score, stromal score, and ME score. Analyses were implemented with R version 4.0.3. Wilcox test was performed to test for significant differences between the two groups. *P* < 0.05 was considered statistically significant.

### 2.6. Expression of the PDGF Family in Stromal and Epithelial Components of Ovarian Cancer

The mRNA expression profiles of ovarian cancer GSE40595 were downloaded from the Gene Expression Omnibus (GEO, https://www.ncbi.nlm.nih.gov/geo/) database, including 30 microdissected tumor stromal samples and 32 microdissected tumor epithelial samples. The statistical difference in the expression of the PDGF family genes between tumor stromal and epithelial components was compared by the Wilcox test.

### 2.7. Correlation Analysis of PDGF Family Expression with CAFs in Ovarian Cancer and Pancancer

The association between the individual gene expression of PDGF family members and CAFs infiltration in ovarian cancer and pancancer was analyzed by the Tumor Immune Estimation Resource Version 2 (TIMER2.0, http://timer.cistrome.org/) using EPIC, MCPcounter, XCELL, and TIDE algorithms. We selected the Purity Adjustment option to avoid the major confounding factor of tumor purity. The correlation was evaluated using the Spearman's correlation. *P* < 0.05 was considered statistically significant.

### 2.8. Correlation Analysis of PDGF Family Expression and Macrophage Infiltration in Ovarian Cancer and Pancancer

The association of PDGF family expression with the immune scores of various tumor-infiltrating immune cells in 376 TCGA ovarian cancer samples was obtained by Spearman's correlation. *P* < 0.05 was considered statistically significant. The association between the individual gene expression of PDGF family members and macrophage infiltration in ovarian cancer was obtained from TIMER2.0 using TIMER algorithm by Spearman's correlation. Purity Adjustment option was used to avoid the major confounding factor of tumor purity. *P* < 0.05 was considered statistically significant.

To assess the correlation between the PDGF family and immune score of macrophages M0, M1, and M2 in pancancer, we used the reliable immunodeconv R software package which integrates six latest algorithms, including Timer, Xcell, MCPcounter, Ciberport, Epic, and Quantiseq. All analysis methods and R package were implemented by R version 4.0.3. The significant differences between the two groups were analyzed by the Wilcox test. *P* < 0.05 were considered statistically significant.

### 2.9. Prognostic Analysis of the PDGF Family in Ovarian Cancer

Overall survival (OS), progression-free survival (PFS), and postprogression survival (PPS) were calculated using the Kaplan–Meier plotter method (http://kmplot.com/), and statistical significance was assessed using the log-rank test. *P* < 0.05 were considered statistically significant.

### 2.10. Construction of PDGF Family-Based Risk Prognosis Model of Ovarian Cancer

RNA-sequencing expression profiles of 111 patients with ovarian cancer and complementary prognostic information were downloaded from the International Cancer Genome Consortium (ICGC, https://dcc.icgc.org/releases/current/Projects) dataset. The least absolute shrinkage and selection operator (LASSO) is a regression algorithm that introduces a regularization penalty factor lambda on the regression weights to filter uninformative and avoid overfitting using 10-fold cross-validation. The LASSO regression was used to select highly relevant variables from the PDGF family members. And the R software package (v 4.1-1) was used for the analysis. The coefficients of selected features were shown by the lambda parameter. The optimal lambda value was selected by the least partial likelihood deviance in the LASSO method. When lambda is the smallest, the model reaches the best. Multivariate Cox regression analysis was performed to construct a prognosis model. Subsequently, the step function was performed during the iteration, and the optimal model was selected as the final model. For Kaplan-Meier curves of OS in ovarian cancer stratified by PDGF family-based signature in high- and low-risk groups, *P* values and hazard ratio (HR) with 95% confidence interval (CI) were generated by log-rank tests and univariate Cox proportional hazards regression. Log-rank test was used to compare differences in survival between selected groups. ROC analysis was performed to compare the predictive accuracy of the risk score based on the selected signature. All analysis methods and R packages were implemented by R version 4.0.3. *P* value < 0.05 was considered statistically significant.

## 3. Results

### 3.1. The PDGF Family Expressed Differentially in Ovarian Cancer

**Figure 1:**
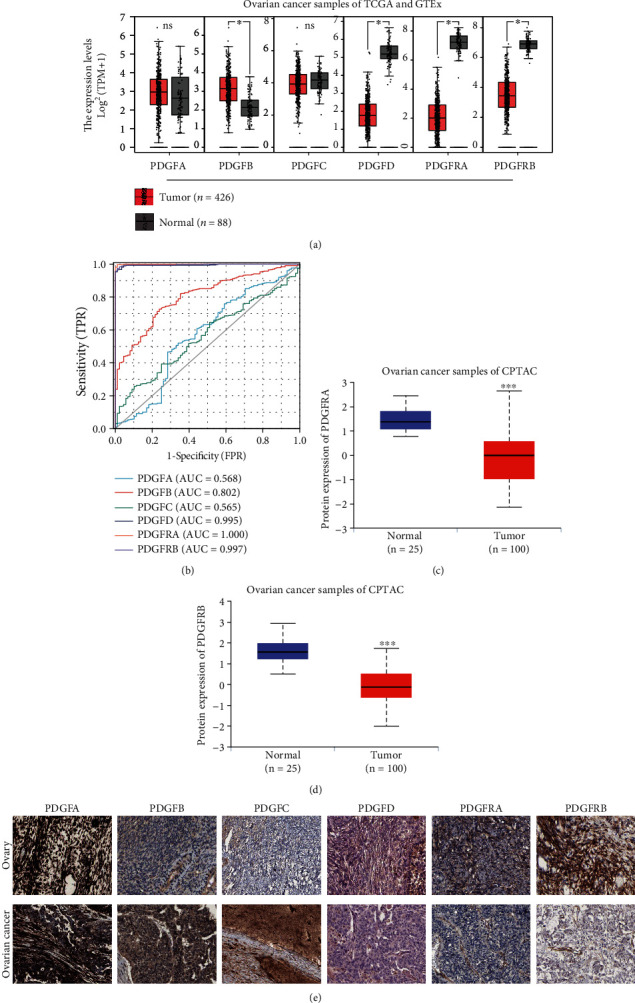
Expression of the PDGF family in ovarian cancer. (a) The mRNA levels of the PDGF family genes in 426 ovarian cancer samples of the TCGA database and 88 normal ovarian samples of the GTEx dataset. ^∗^ or ^∗∗∗^: significantly different from the corresponding normal control, *P* < 0.05 or *P* < 0.001, respectively; ns: not significantly different from the corresponding control (Wilcoxon rank-sum test). (b) ROC analysis of the PDGF family in predicting the sample state (normal or tumor) of ovarian cancer. (c–d) The total protein level of PDGFRA (c) and PDGFRB (d) in 25 normal ovarian samples and 100 ovarian cancer samples from the CPTAC dataset. ^∗∗∗^: significantly different from the normal group, *P* < 0.001 (Wilcox test). (e) Representative images of PDGF family IHC staining in the ovary and ovarian cancer from the HPA database.

To detect the expression of the PDGF family in ovarian cancer, we first query the expression data in ovarian cancer from the TCGA database and normal ovarian data from the GTEx database. As apparent from Figure 1(a), the mRNA level of PDGFB was moderately increased in tumor tissues of ovarian cancer in comparison to their normal counterparts, whereas the mRNA expression levels of PDGFD, PDGFRA, and PDGFRB were markedly decreased in ovarian cancer tissues. ROC analysis also demonstrated that PDGFD (AUC = 0.995), PDGFRA (AUC = 1.000), and PDGFRB (AUC = 0.997) could be a single significant parameter to discriminate between normal and tumor tissues of ovarian cancer. However, the predictive ability was certain for the variable PDGFB (AUC = 0.802), low for PDGFA (AUC = 0.568) and PDGFC (AUC = 0.565) (Figure 1(b)). By virtue of UALCAN analysis, we identified that the protein levels of PDGFRA and PDGFRB were significantly lower in tumor tissues of ovarian cancer than those in normal tissues (Figures 1(c) and 1(d)). Consistently, more intense IHC staining of PDGFRA and PDGFRB was observed in normal ovarian tissues than in tumor tissues of ovarian cancer. Notably, positive staining for PDGF receptors was mainly observed in ovarian cancer tissues' tumor stroma. In parallel with the mRNA expression, the staining of PDGFB in ovarian cancer tissues was higher compared to normal tissues, and PDGFB was predominantly expressed by tumor cells. However, there were no apparent differences in the protein expression of PDGFA and PDGFC in tumor tissues of ovarian cancer and normal ovarian tissues. Intriguingly, the expression levels of PDGF ligands in tumor cells were inconsistent, as PDGFA and PDGFB stained strongly, whereas PDGFC and PDGFD stained weakly in ovarian cancer tumor cells (Figure 1(e)). Furthermore, analyses of PDGFRA and PDGFRB expression in pancancer tissues additionally demonstrated that the reduction of PDGFRA and PDGFRB at both mRNA and protein levels in tumor tissues might be a common event, as elucidated by previous studies [12] (Figure S1).

### 3.2. The PDGF Family Was Associated with ECM Organization and Remodeling in Ovarian Cancer

**Figure 2:**
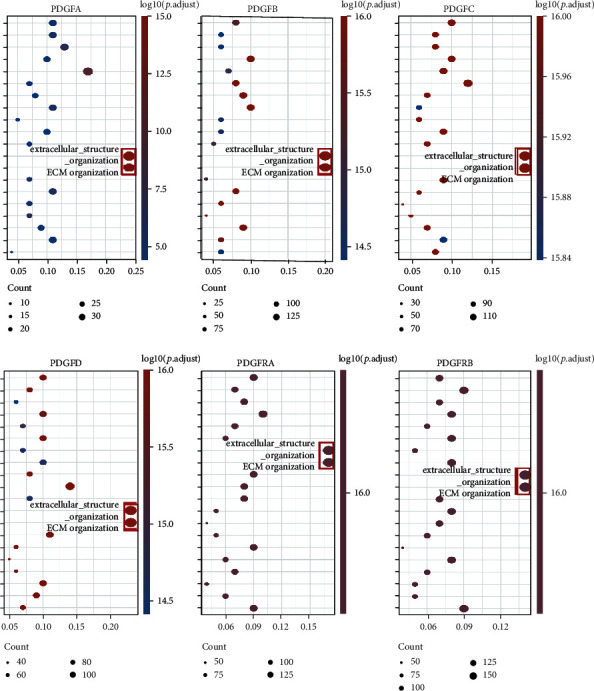
GO analysis of the PDGF family in ovarian cancer of the TCGA database. Circle colors represent the significance of differential enrichment, and circle sizes denote the number of enriched genes in the respective category.

To better decipher the biological processes and rationale of the PDGF family in ovarian cancer, GO and KEGG enrichment analysis was performed. First, ovarian cancer samples of the TCGA database were dichotomized based on the individual expression level of the PDGF family members. DEGs between the high- and low-expression groups was obtained using the limma package. As summarized in Figure S2, we observed that the most upregulated DEGs in the high-expression group were strongly positively correlated with ECM composition-related genes (e.g., COL1A1/2, COL3A1, COL5A1/2, COL6A2/3, COL8A1, COL10A1, COL11A1, FN1, VCAN, DCN, THBS2, LUM, POSTN, and EMILIN1) and ECM remodeling-related genes (MMP2/11, SFRP2/4, LRRC15, AEBP1, TIMP3, COMP, MFAP4, PTGIS, and FNDC1). In addition, from the bubble plots of the GO analysis, it is apparent to see that for each PDGF family member, the majority of genes in the high-expression group were significantly enriched in pathways related to ECM, including “ECM organization” and “extracellular structure organization” (Figure 2). Other highly enriched pathways were displayed in Figure S3, such as “connective tissue development,” “collagen metabolic process,” “collagen fibril organization,” and “cell-substrate adhesion,” all involved in the organization of ECM [19]. Meanwhile, KEGG analysis also showed that the genes in the high-expression group for all PDGF family members were also relevant to “ECM-receptor interaction” and “proteoglycans in cancer,” which were required for ECM composition and remodeling (Figure S4) [20]. All the above results revealed the crucial involvement of the PDGF family in the ECM organization of ovarian cancer.

**Figure 3:**
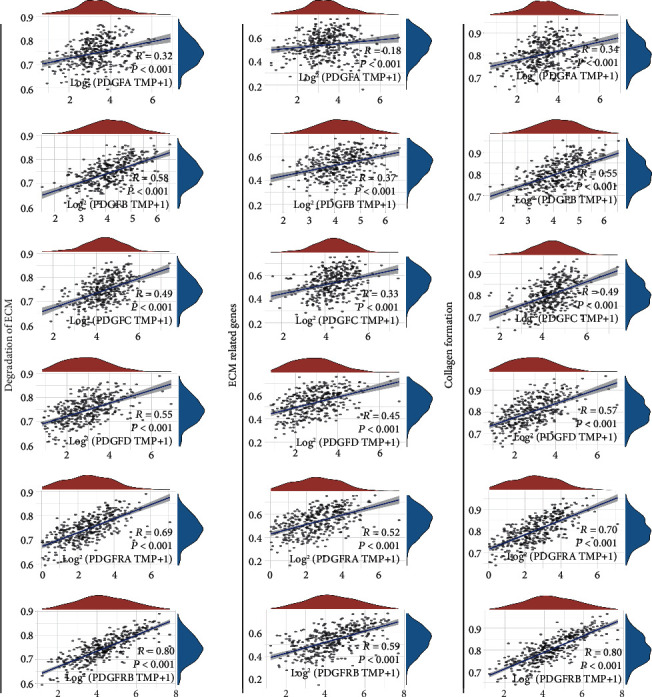
Correlation analysis of individual gene expression of the PDGF family with pathway scores in ovarian cancer. The correlation between gene expression and the pathway was evaluated by calculating Spearman's correlation coefficient (*R*).

Similar findings were also observed in the correlation analysis of all PDGF family members in 376 TCGA ovarian cancer samples with pathway scores using the R software GSVA package and chose parameters as a method “ssgsea” (Figure 3). For each gene of the PDGF family, a positive correlation was observed with “degradation of ECM” (*R* = 0.32 for PDGFA, *R* = 0.58 for PDGFB, *R* = 0.49 for PDGFC, *R* = 0.55 for PDGFD, *R* = 0.69 for PDGFRA, and *R* = 0.80 for PDGFRB; *P* < 0.001 for all), “ECM-related genes” (*R* = 0.18 for PDGFA, *R* = 0.37 for PDGFB, *R* = 0.33 for PDGFC, *R* = 0.45 for PDGFD, *R* = 0.52 for PDGFRA, and *R* = 0.59 for PDGFRB; *P* < 0.001 for all), and “collagen formation” (*R* = 0.34 for PDGFA, *R* = 0.55 for PDGFB, *R* = 0.49 for PDGFC, *R* = 0.57 for PDGFD, *R* = 0.70 for PDGFRA, and *R* = 0.80 for PDGFRB; *P* < 0.001 for all). In particular, the correlation coefficients of PDGFRA and PDGFRB were upmost among all members (Figure 3).

### 3.3. The PDGF Family Was Predominantly Expressed in the Tumor Stroma of Ovarian Cancer

**Figure 4:**
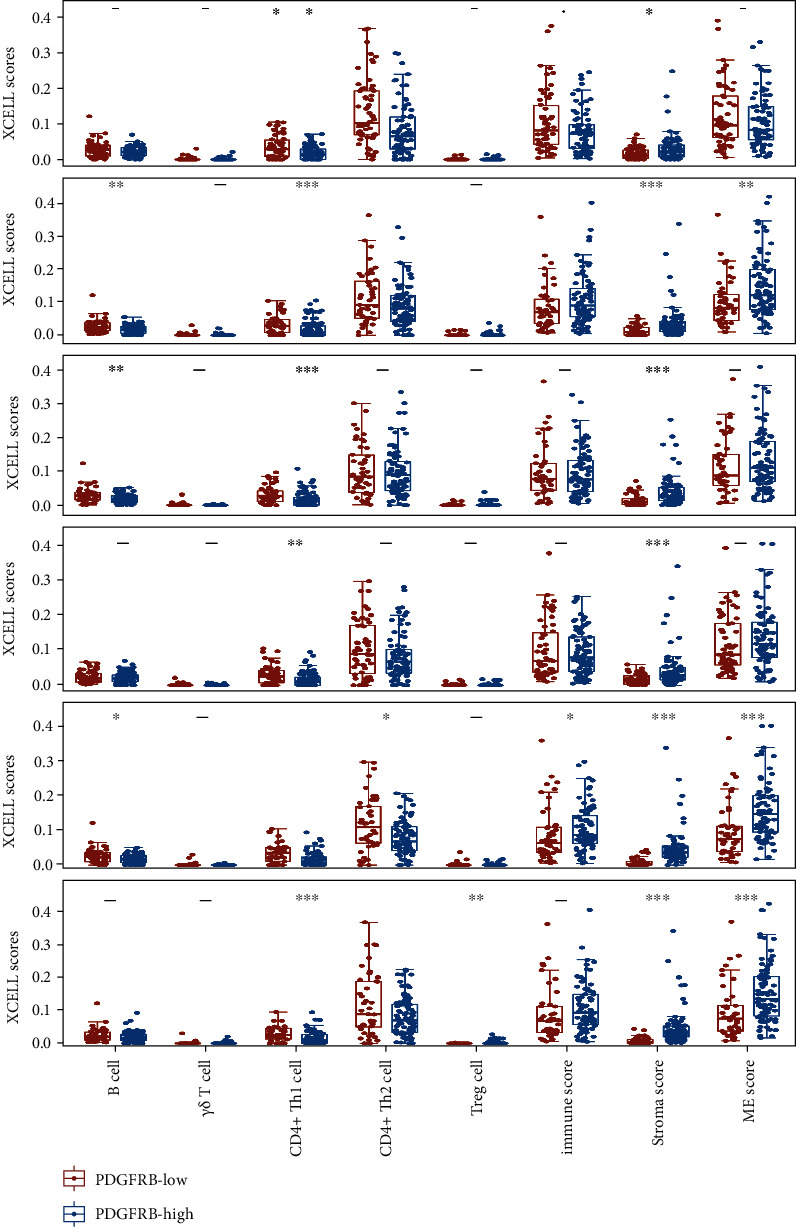
The differences in XCELL scores of microenvironmental variables between the individual high- and low-expression groups of the PDGF family. ^∗^, ^∗∗^, ^∗∗∗^: significantly different from the respective low-expression group, *P* < 0.05, *P* < 0.01, and *P* < 0.001, respectively (Wilcox test).

Based on the above findings, we speculated the tight connection of the PDGF family in ECM organization might at least partially relate to the expression of the PDGF family dominated by stromal components of ovarian cancer. To test this hypothesis, we focused on the expression pattern of the PDGF family in ovarian cancer. Firstly, we assessed the correlation of the PDGF family with ME variables, including the abundance of tumor-infiltrating immune cells, immune score, stromal score, and ME score using the mRNA expression data of 376 TCGA ovarian cancer samples. The distributions of different ME variables between the high- and low-expression groups of each gene of the PDGF family are shown in Figure 4. More specifically, all stroma scores in the high-expression group of the PDGF family genes were significantly higher compared to the respective low-expression groups. And we also noticed that the ME scores were higher in the PDGFRA-high group and the PDGFRB-high group than those in the respective low-expression groups, respectively (Figure 4). It must be emphasized that, although statistically significant, the differences in terms of stroma scores and ME scores were relatively weak to draw robust conclusions that the PDGF family was predominantly expressed in the tumor stroma of ovarian cancer.

**Figure 5:**
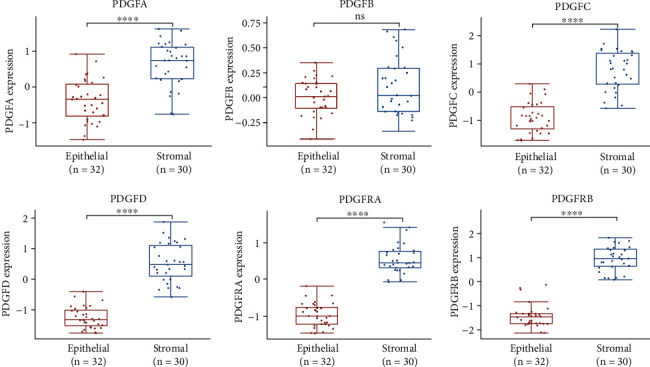
The individual mRNA expression level of the PDGF family in epithelial and stromal components of ovarian cancer. ^∗∗∗∗^ significantly different from the epithelial group, *P* < 0.0001; ns: not significantly different from the epithelial group (Wilcox test).

Since the expression profiles of the TCGA database were generated from bulk tumor samples with varying degrees of stromal infiltration, rendering us unable to distinguish the expression differences of the PDGF family between tumor cells and tumor stroma. Hence, we subsequently turn to the GSE40595 from the GEO database, including 30 microdissected stromal samples and 32 epithelial samples of ovarian cancer tissues. Except for PDGFB, the rest six genes of the PDGF family were substantially increased in the stromal components of ovarian cancer compared with epithelial components (Figure 5). Even though the difference in PDGFB expression between the two groups was not statistically significant, we can still observe a moderate increase of PDGFB in ovarian cancer stromal components. These data indicated that the PDGF family was mainly expressed by tumor stromal components of ovarian cancer.

### 3.4. The PDGF Family Was Involved in Tumor Metastasis of Ovarian Cancer

**Figure 6:**
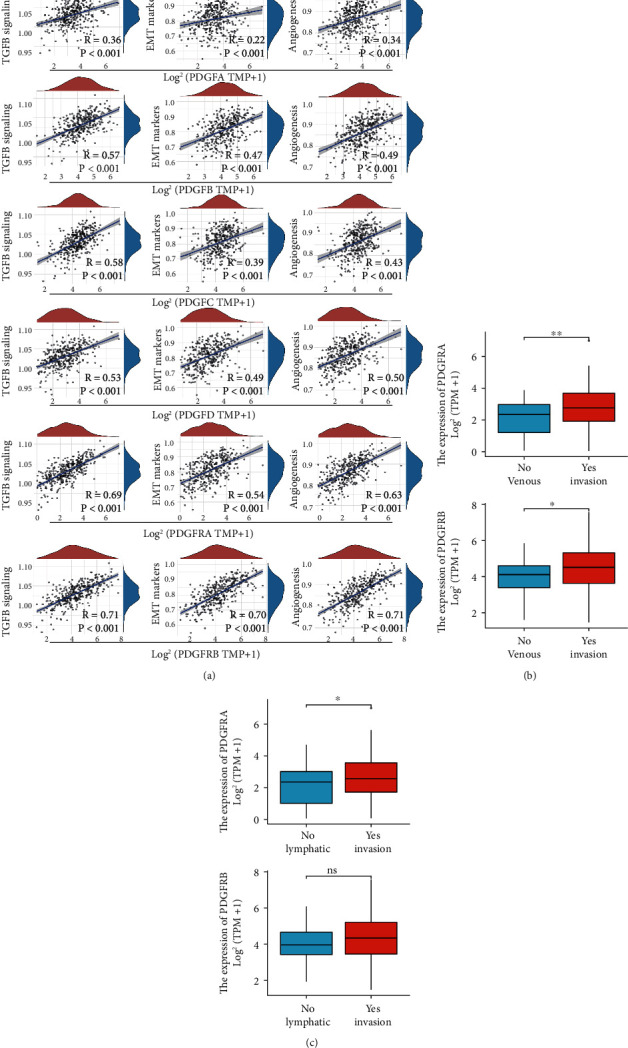
The PDGF family was involved in tumor metastasis of ovarian cancer. (a) Correlation analysis of individual gene expression of the PDGF family with pathway scores in ovarian cancer. The correlation between gene expression and the pathway was evaluated by Spearman's correlation coefficient (*R*). (b) The mRNA expression levels of PDGFRA and PDGFRB in ovarian cancer patients with or without venous invasion. (c) The mRNA expression levels of PDGFRA and PDGFRB in ovarian cancer patients with or without lymphatic invasion. ^∗^, ^∗∗^: significantly different from the corresponding control group, *P* < 0.05, *P* < 0.01, respectively; ns: not significantly different from the corresponding control group (Wilcox test).

Considering that ECM governed the aggressiveness and metastasis in multiple cancer types by mediating cell proliferation, migration, differentiation, and adhesion [19], we then investigated whether the PDGF family was linked to the key biological processes suggestive of oncogenic properties. As expected, we noticed that all PDGF family members were intimately associated with transforming growth factor *β* (TGF-*β*) signaling (*R* = 0.36 for PDGFA, *R* = 0.57 for PDGFB, *R* = 0.58 for PDGFC, *R* = 0.53 for PDGFD, *R* = 0.69 for PDGFRA, and *R* = 0.71 for PDGFRB; *P* < 0.001 for all), epithelial-mesenchymal transition (EMT) (*R* = 0.22 for PDGFA, *R* = 0.47 for PDGFB, *R* = 0.39 for PDGFC, *R* = 0.49 for PDGFD, *R* = 0.54 for PDGFRA, and *R* = 0.70 for PDGFRB; *P* < 0.001 for all), and angiogenesis (*R* = 0.34 for PDGFA, *R* = 0.49 for PDGFB, *R* = 0.43 for PDGFC, *R* = 0.50 for PDGFD, *R* = 0.63 for PDGFRA, and *R* = 0.71 for PDGFRB; *P* < 0.001 for all), which are all involved in tumor metastasis (Figure 6(a)) [21, 22]. KEGG analysis of the PDGF family in ovarian cancer showed that all members were closely connected in “phosphatidylinositol 3-kinase-Akt (PI3K-Akt) signaling” (Figure S4), which is notoriously related to tumorigenesis and invasiveness in cancers, including ovarian cancer [23]. Notably, for PDGFRA and PDGFRB, epithelial cell proliferation, epithelial cell migration, positive regulation of cell adhesion, and angiogenesis were also highly enriched (Figure S3), suggesting that PDGFRA and PDGFRB might be critically involved in the metastasis of ovarian cancer. Furthermore, the mRNA levels of PDGFRA and PDGFRB in ovarian cancer patients with venous invasion were higher than those in patients without venous invasion. Besides, ovarian cancer patients with lymphatic invasion expressed higher mRNA levels of PDGFRA and PDGFRB, even though the difference in PDGFRB had no statistical significance. Overall, the PDGF family was capable of mediating tumor invasiveness and metastasis of ovarian cancer.

### 3.5. The PDGF Family Was Associated with CAF Infiltration in Ovarian Cancer and Pancancer

**Figure 7:**
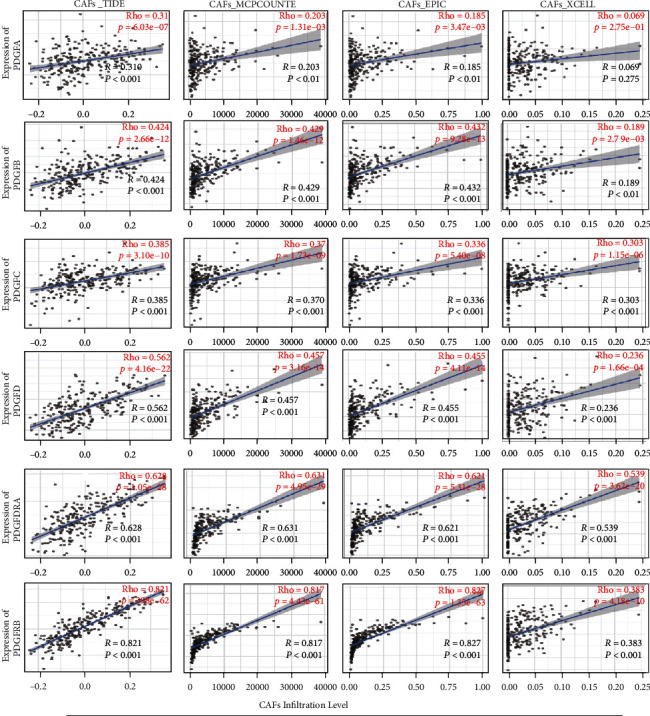
Correlation analysis of PDGF family expression with CAFs infiltration level in ovarian cancer. The correlation between gene expression and CAF infiltration was evaluated by calculating Spearman's correlation coefficient (*R*).

Since CAFs are the most abundant constituents of tumor stroma and are regarded as a hallmark of cancer [8], we next evaluated the association between the PDGF family and CAF infiltration. The correlation analysis revealed that the individual gene expression of the PDGF family was broadly positively associated with CAF infiltration in ovarian cancer according to TIDE (*R* = 0.310 for PDGFA; *R* = 0.424 for PDGFB; *R* = 0.385 for PDGFC; *R* = 0.562 for PDGFD; *R* = 0.628 for PDGFRA; *R* = 0.821 for PDGFRB; all *P* < 0.001), MCPcounter (*R* = 0.203 and *P* < 0.01 for PDGFA; *R* = 0.429 and *P* < 0.001 for PDGFB; *R* = 0.370 and *P* < 0.001 for PDGFC; *R* = 0.457 and *P* < 0.001 for PDGFD; *R* = 0.631 and *P* < 0.001 for PDGFRA; *R* = 0.817 and *P* < 0.001 for PDGFRB), and EPIC (*R* = 0.185 and *P* < 0.01 for PDGFA; *R* = 0.432 and *P* < 0.001 for PDGFB; *R* = 0.336 and *P* < 0.001 for PDGFC; *R* = 0.455 and *P* < 0.001 for PDGFD; *R* = 0.621 and *P* < 0.001 for PDGFRA; *R* = 0.827 and *P* < 0.001 for PDGFRB), and XCELL (*R* = 0.069 and *P* = 0.275 for PDGFA; *R* = 0.189 and *P* < 0.01 for PDGFB; *R* = 0.303 and *P* < 0.001 for PDGFC; *R* = 0.236 and *P* < 0.001 for PDGFD; *R* = 0.539 and *P* < 0.001 for PDGFRA; *R* = 0.383 and *P* < 0.001 for PDGFRB) (Figure 7).

**Figure 8:**
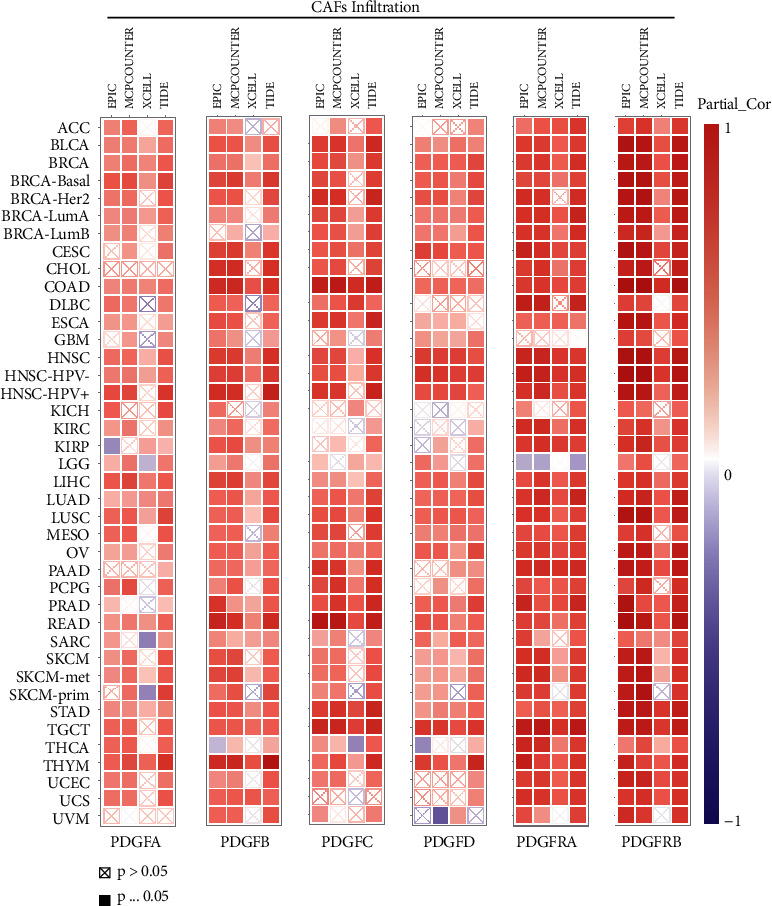
Correlation analysis of PDGF family expression with CAFs infiltration in pancancer. The correlation between gene expression and CAFs infiltration was evaluated according to Spearman's correlation coefficient (*R*). The color of each square block represents the strength of the correlation, with red indicating strong positive correlation, blue indicating negative correlation, and white indicating no relatedness.

Furthermore, the correlation analysis was further performed in pancancer, also showing a strong association of the PDGF family with CAF infiltration in practically all cancer types according to EPIC, MCPcounter, XCELL, and TIDE algorithms. Especially for PDGFRA and PDGFRB, there existed a strong positive association with CAF infiltration (Figure 8).

### 3.6. The PDGF Family Was Associated with Tumor-Associated Macrophage (TAM) Infiltration in Ovarian Cancer and Pancancer

**Figure 9:**
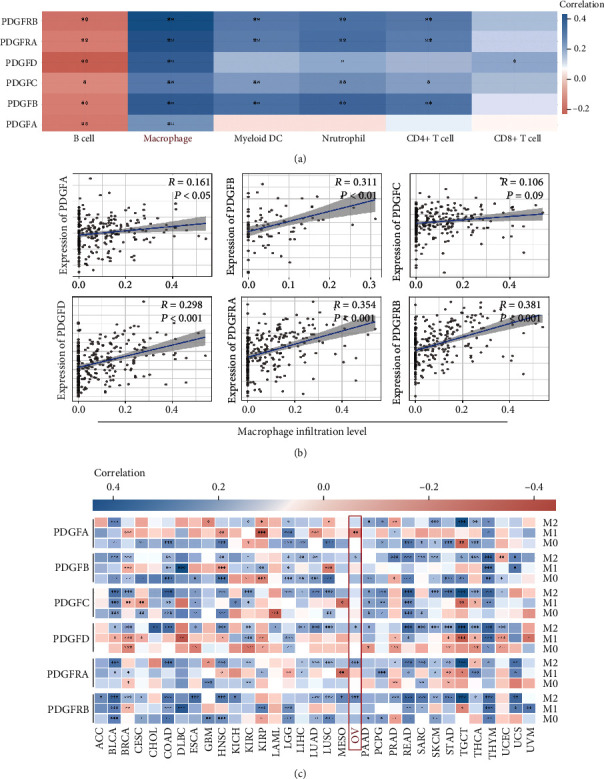
The PDGF family was associated with macrophage infiltration in ovarian cancer and pancancer. (a) Correlation of PDGF family expression with immune scores of six immune cell subtypes. (b) Spearman's correlation of PDGF family expression with macrophage infiltration in ovarian cancer. (c) Spearman's correlation heatmap between macrophage (M0, M1, and M2) infiltration and the individual mRNA expression of the PDGF family in tumor tissues of multiple cancer types. The color of each square block represents the strength of the correlation, with blue indicating positive correlation, red indicating negative correlation, and white indicating no relatedness. The darker the color, the stronger the correlation. ^∗^*P* < 0.05, ^∗∗^*P* < 0.01, ^∗∗∗^*P* < 0.001 (Wilcox test).

Given that macrophages were the most prominent immune cells in the tumor stroma, referred to as tumor-associated macrophages (TAMs) [24], it is necessary to investigate the relationship between PDGF family expression and TAM infiltration in ovarian cancer. Thus, we evaluated the correlation of the PDGF family expression with the immune scores of six tumor-infiltrating immune cell subtypes. The results showed that the expression levels of all PDGF family members were positively associated with macrophage infiltration but negatively correlated with B cell infiltration (Figure 9(a)). In addition, via the TIMER2.0, we obtained the positive association of macrophage infiltration levels with the mRNA levels of PDGFA (*R* = 0.161, *P* < 0.05), PDGFB (*R* = 0.311, *P* < 0.01), PDGFC (*R* = 0.106, *P* = 0.09), PDGFD (*R* = 0.298, *P* < 0.001), PDGFRA (*R* = 0.354, *P* < 0.001), and PDGFRB (*R* = 0.381, *P* < 0.001) (Figure 9(b)). Based on global gene expression patterns, TAMs are categorized into three distinct subtypes, M0 (undifferentiated), M1 (anti-tumor), and M2 (tumor-promoting) [25]. Hence, we generated the relationship of the PDGF family expression with the infiltration of macrophages M0, M1, and M2 in pancancer. In ovarian cancer, PDGFB (*P* < 0.05), PDGFD (*P* < 0.05), PDGFRA (*P* < 0.001), and PDGFRB (*P* < 0.001) had a significant positive correlation with macrophage M2 infiltration, and PDGFA had a negative association with macrophage M1 infiltration (*P* < 0.01). Moreover, in bladder urothelial carcinoma (BLCA), testicular germ cell tumors (TGCT), skin cutaneous melanoma (SKCM), and thymoma (THYM), there also existed a positive correlation between macrophage M2 infiltration levels with all gene expression levels of the PDGF family (Figure 9(c)).

### 3.7. Construction of the PDGF Family-Based Risk Prognosis Model in Ovarian Cancer

It has long been assumed that tumor stroma activation is considered an essential determinant of tumor aggressiveness [6], which notoriously predicates poor survival in patients with various cancer types, including ovarian cancer [10, 26–29]. To evaluate the predictive value of the PDGF family in ovarian cancer, we first performed the Kaplan-Meier plotter analysis. The results showed a higher expression of PDGFA and PDGFB in ovarian cancer related to a poorer clinical survival (worse OS and PPS for PDGFA, worse PPS for PDGFB). Despite lower expression in ovarian cancer than in normal tissues, patients with higher PDGFD, PDGFRA, and PDGFRB also had worse OS, PFS, and PPS in ovarian cancer (Table 1). The above observations suggested that higher expression of the PDGF family predicated an unfavorable clinical survival for patients with ovarian cancer.

**Figure 10:**
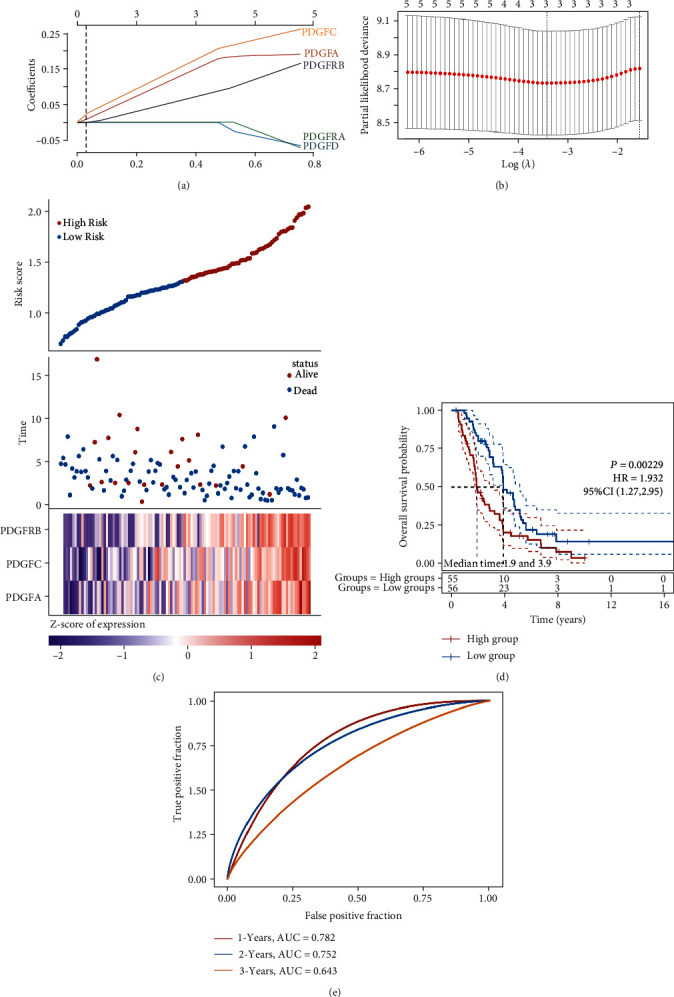
Construction of the PDGF family-based risk prognosis model in ovarian cancer. (a) The relationship between lambda values and coefficients. The abscissa represents the value of lambda, and the ordinate represents the coefficients of the independent variable. (b) The partial likelihood deviance was plotted versus log (*λ*) using the LASSO Cox regression model. (c) The distribution of risk score, survival time, survival status, and expression panel of PDGFA, PDGFC, and PDGFRB in ovarian cancer. The top scatter plot indicates the risk score from low to high. The middle represents the scatter plot distribution of survival time and survival status corresponding to the risk score of PDGFA, PDGFC, and PDGFRB. The bottom is the heatmap of PDGFA, PDGFC, and PDGFRB expression in ovarian cancer. (d) Kaplan-Meier survival curve of OS in ovarian cancer classified by PDGF family-based signature in high- and low-risk groups based on the risk score. (e) The ROC curve of the selected genes. The higher values of AUC correspond to higher predictive power for clinical survival prognosis of ovarian cancer patients.

Then, we applied the LASSO Cox regression to establish a prognosis model based on the PDGF family in 111 patients with ovarian cancer derived from the ICGC dataset. Among the seven genes, PDGFA, PDGFC, and PDGFRB were selected as the optimal predictors in the model and were defined as the PDGF family-based signature for the prognosis of ovarian cancer (Figure 10(a)). The one with minimal average deviance (lambda.min = 0.0317) was set as the best lambda value by 10-fold cross-validation (Figure 10(b)). The risk formula was obtained with the expression levels of the three genes and the respective regression coefficients: risk core = 0.1671 × PDGFA + 0.1942 × PDGFC + 0.0832 × PDGFRB. The expression panels of the three genes in ovarian cancer and the corresponding risk score, survival time, and survival status are shown in Figure 10(c). It was apparent that higher expression levels of PDGFA, PDGFC, and PDGFRB were correlated with higher risk scores (Figure 10(c)). The median risk score was taken as the cut-off value. According to the optimal risk cut-off point, 111 patients were stratified by the PDGF family-based signature into high- and low-risk groups. As expected, the Kaplan-Meier survival analyses of OS in ovarian cancer uncovered that the patients in the high-risk group showed a significantly worse OS in comparison with the low-risk group (Figure 10(d), hazard ratio (HR) = 1.932, 95 confidence interval (CI) = 1.27–2.95, *P* < 0.01). Moreover, a time-dependent ROC curve was performed to predict the 1-, 2-, and 3-year survival rates, showing that the risk prognosis model had high prediction accuracy, especially for 1 year (AUC = 0.782) and 2 years (AUC = 0.752) survival rates (Figure 10(e)). Overall, these findings confirmed that the PDGF family based-signature was a risk factor for patients with ovarian cancer.

## 4. Discussion

It has been extensively accepted that the occurrence and progression of cancer result from genetic alterations intrinsic to cancer cells as well as the mutual communication between cancer cells and the surrounding tumor stroma [5]. It is acknowledged that tumor stroma can produce growth factors, cytokines, and chemokines to sustain the oncogenic capacity of cancer cells; conversely, a series of signals originating from cancer cells also sustained the nutrient stroma conducive to the pathological development of ovarian cancer [6]. Not surprisingly, mounting evidence recognized the ratio of tumor to stroma as an indicator for the clinical survival outcome in such epithelial cancer types as esophageal squamous cancer [30], breast cancer [26], colon cancer [29], cervical cancer [28], and ovarian cancer [10, 27, 31]. In addition, tumor stroma was reported to be associated with peritoneal metastasis [9], hematogenous and lymphatic metastasis [32], immune response, and chemotherapy [11].

PDGF ligands, encoded by PDGFA, PDGFB, PDGFC, and PDGFB, are regarded as potent mitogens and chemoattractants for mesenchymal cells through interacting with PDGF receptors (encoded by PDGFRA and PDGFRB) [12]. PDGF receptors are commonly expressed by stromal cells [15], indicating that PDGF may participate in tumorigenesis via the paracrine form. In line with previous reports [12, 14], we observed that PDGFRA and PDGFRB were predominantly expressed in the tumor stroma of ovarian cancer but merely expressed in cancer cells. Compared with the respective normal tissues, the mRNA and protein levels of PDGFRA and PDGFRB were substantially lower in tumor tissues of almost all cancer types, including ovarian cancer. In addition, our findings also showed that higher expression levels of the PDGF family members were observed in stromal components in comparison with epithelial components and were associated with higher stroma scores. The association of the PDGF family with tumor stroma indicated that the PDGF family might participate in tumor progression by affecting the tumor stroma of ovarian cancer.

After binding to PDGF receptors, PDGF ligands stimulate the intracellular signal cascades, e.g., PI3K-Akt, Janus kinase (JAK), mitogen-activated protein kinase/extracellular signal-regulated protein kinase (MAPK/ERK), thereby to promote tumor cell proliferation and invasion [33]. Through interrogating the biological function of the PDGF family in ovarian cancer, we uncovered a strong correlation of the PDGF family with pathways related to degradation and remodeling of the ECM, facilitating tumor invasion and metastasis [19]. As expected, our results showed that the PDGF family was highly connected to tumor-promoting pathways, including TGF-*β* signaling, EMT markers, angiogenesis, and PI3K/Akt signaling [21–23]. Besides, the expression levels of PDGFRA and PDGFRB were significantly associated with venous invasion and lymphatic invasion of ovarian cancer. As the most abundant component in the tumor stroma, CAFs participate in tumor growth, migration, invasion, angiogenesis, lymphangiogenesis, and therapy resistance of ovarian cancer by altering the biological properties and oncogenic capacities of cancer cells [34, 35]. Among the various growth factors involved in CAF recruitment and differentiation, TGF-*β* and PDGF are generally considered the most important [36]. Here, we found a strong correlation between the PDGF family with CAFs infiltration not only in ovarian cancer but in a variety of cancers. It is also worth noting that the PDGF family was positively correlated with macrophage infiltration, especially macrophage M2, in ovarian cancer and pancancer, which is perceived as tumor-promoting and predicates poor clinical prognosis in patients with ovarian cancer [25]. From the findings revealed in the above analyses, it could conceivably be hypothesized that the metastasis and progression of ovarian cancer were not only due to the enhanced oncogenic capacity of cancer cells but also due to the activation of tumor stroma, including the recruitment of CAFs and TAMs and remodeling of the ECM.

One study previously identified the PDGF signaling pathway as a powerful biomarker that significantly stratifies the survival rates of patients with ovarian cancer from the TCGA database [37]. Given the fact that a higher abundance of tumor stroma predicted a poor prognosis in patients with ovarian cancer [10, 27, 31], we further evaluated the performance of the PDGF family-based signature for predicting clinical survival in patients with ovarian cancer. Using the LASSO Cox regression, a three-gene (PDGFA, PDGFC, and PDGFRB)-based signature was constructed, and the robustness of this signature was well validated in the cohorts of the ICGC dataset by the Kaplan-Meier survival analyses of OS (HR = 1.932, *P* < 0.01; AUC = 0.782, 0.752 for 1 year and 2 years, respectively). Thus, the PDGF family-based signature could act as a determiner of prognosis for patients with ovarian cancer. One of the main facts that cause difficulty in treating ovarian cancer is the high rate of therapy resistance despite the initial response to platinum-based chemotherapy. Due to the genetic stability of tumor stroma relative to cancer cells, novel approaches selectively targeting the tumor stroma are increasingly recognized as a practical approach by abrogating the tumor-stroma interplay [7, 38]. There are already several preclinical studies on tumor stroma targeting strategies, such as inhibition of TGF-*β* signaling, fibroblast growth factor (FGF) pathway, focal adhesion kinase (FAK), and CXC-chemokine receptor 4 [8]. An implication of the tight association of the PDGF family with tumor stroma and the poor prognosis is the possibility that patients with high PDGF family expression may be more suitable for stromal-targeted therapy. The combination of our findings, while preliminary, provides some support for the likelihood of application of PDGF family-based tumor stroma targeting therapies as mentioned in the literature [15].

However, there still exist some limitations in our study. First, all analyses were obtained through data mining of public databases without validation through our clinical samples or fundamental experiments. Second, the specific molecular mechanism of the effect of the PDGF family on stroma activation and tumor metastasis is not available and needs in-depth exploration, which is also our follow-up work. Furthermore, the retrospective samples and the small number of cases (*n* = 111) in our studied cohort reminded us that we should acknowledge the limitations of our risk prognosis model. More prospective cohorts should be recruited to validate the robustness and stability of PDGF family-based signature to predicate survival outcomes in ovarian cancer patients.

## 5. Conclusions

In conclusion, our findings reveal that ovarian cancers with high PDGF family expression biologically resemble metastatic tumors and exhibit a poor prognosis, which is attributed to the activated tumor stroma in ovarian cancers.

## Figures and Tables

**Table 1:** Prognostic analysis of the PDGF family in ovarian cancer.

Gene	OS^1^		PFS^2^		PPS^3^	
	HR^4^	*P* value	HR	*P* value	HR	*P* value
PDGFA	1.18 (1.03-1.36)	<0.05	0.87 (0.75-1.00)	0.053	1.28 (1.09-1.52)	<0.01
PDGFB	0.94 (0.81-1.08)	0.38	0.88 (0.78-1.00)	0.053	1.21 (1.02-1.43)	<0.05
PDGFC	1.1 (0.95-1.26)	0.19	1.2 (1.06-1.37)	<0.01	1.16 (0.98-1.38)	0.077
PDGFD	1.3 (1.14-1.48)	<0.001	1.35 (1.19-1.53)	<0.001	1.23 (1.02-1.49)	<0.05
PDGFRA	1.32 (1.14-1.53)	<0.001	1.34 (1.16-1.56)	<0.001	1.31 (1.08-1.6)	<0.01
PDGFRB	1.24 (1.09-1.41)	<0.001	1.28 (1.12-1.46)	<0.001	1.46 (1.22-1.74)	<0.001

^1^Overall survival. ^2^Progression-free survival. ^3^Postprogression survival. ^4^Hazard Ratio.

## Data Availability

All public datasets used in our study can be found on http://cancergenome.nih.gov/, https://www.ncbi.nlm.nih.gov/geo/, https://www.aclbi.com/, https://www.proteinatlas.org/, http://kmplot.com/, https://www.xiantao.love/, http://timer.cistrome.org/, and http://ualcan.path.uab.edu/analysis-prot.html.

## Abbreviations

ME: Microenvironment
CAF: Cancer-associated fibroblasts
ECM: Extracellular matrix
PDGF: Platelet-derived growth factor
PDGFRA: PDGF receptor *α*
PDGFRB: PDGF receptor *β*
GTEx: Genotype-Tissue Expression
TCGA: The Cancer Genome Atlas
GEPIA: Gene Expression Profiling Interactive Analysis
CPTAC: Clinical Proteomic Tumor Analysis Consortium
UALCAN: University of ALabama at Birmingham Cancer Data Analysis
IHC: Immunohistochemistry
HPA: Human Protein Atlas
ROC: Receiver-operating characteristic
AUC: Area under curve
GO: Gene Ontology
KEGG: Kyoto Encyclopedia of Genes and Genomes
DEGs: Differentially expressed genes
GEO: Gene Expression Omnibus
TIMER2.0: Tumor Immune Estimation Resource Version 2
OS: Overall survival
PFS: Progression-free survival
PPS: Postprogression survival
ICGC: International Cancer Genome Consortium
LASSO: Least absolute shrinkage and selection operator
TGF-*β*: Transforming growth factor *β*
EMT: Epithelial-mesenchymal transition
PI3K-Akt: Phosphatidylinositol 3-kinase-Akt
TAM: Tumor-associated macrophages
BLCA: Bladder urothelial carcinoma
TGCT: Testicular germ cell tumors
SKCM: Skin cutaneous melanoma
THYM: Thymoma
HR: Hazard ratio
CI: Confidence interval
JAK: Janus kinase
MAKP/ERK: Mitogen-activated protein kinase/extracellular signal-regulated protein kinase
FGF: Fibroblast growth factor
FAK: Focal adhesion kinase.

## Supplementary Materials

Supplementary Materials Supplementary Figure S1: the mRNA (a) and protein (b) levels of PDGFRA and PDGFRB in pancancer. ^∗^, ^∗∗^, or ^∗∗∗^: significantly different from the corresponding normal control, *P* < 0.05, *P* < 0.01, or *P* < 0.001, respectively (Wilcoxon rank-sum test). Supplementary Figure S2: the volcano plot of DEGs between the high-expression group and the low-expression group of PDGF family members in ovarian cancer. Red dots indicate upregulated genes, blue dots indicate downregulated genes, and grey dots indicate not significant (high vs. low). Supplementary Figure S3: GO analysis of PDGF family members in ovarian cancer derived from the TCGA database. Circle colors represent the significance of differential enrichment, and circle sizes denote the number of enriched genes in the respective category. Supplementary Figure S4: KEGG analysis of PDGF family members in ovarian cancer derived from the TCGA database. Circle colors represent the significance of differential enrichment, and circle sizes denote the number of enriched genes in the corresponding category.
