# Supplementary material for: The PDGF Family Is Associated with Activated Tumor Stroma and Poor Prognosis in Ovarian Cancer
Source: Dis Markers. 2022 Sep 26;2022:5940049. doi: 10.1155/2022/5940049 (PMC9529473; doi:10.1155/2022/5940049)
Supplement: Supplementary Materials — Supplementary Figure S1: the mRNA (a) and protein (b) levels of PDGFRA and PDGFRB in pancancer. ∗, ∗∗, or ∗∗∗: significantly different from the corresponding normal control, P < 0.05, P < 0.01, or P < 0.001, respectively (Wilcoxon rank-sum test). Supplementary Figure S2: the volcano plot of DEGs between the high-expression group and the low-expression group of PDGF family members in ovarian cancer. Red dots indicate upregulated genes, blue dots indicate downregulated genes, and grey dots indicate not significant (high vs. low). Supplementary Figure S3: GO analysis of PDGF family members in ovarian cancer derived from the TCGA database. Circle colors represent the significance of differential enrichment, and circle sizes denote the number of enriched genes in the respective category. Supplementary Figure S4: KEGG analysis of PDGF family members in ovarian cancer derived from the TCGA database. Circle colors represent the significance of differential enrichment, and circle sizes denote the number of enriched genes in the corresponding category. [file 5940049.f1.zip › Fig S2 (1).pdf]

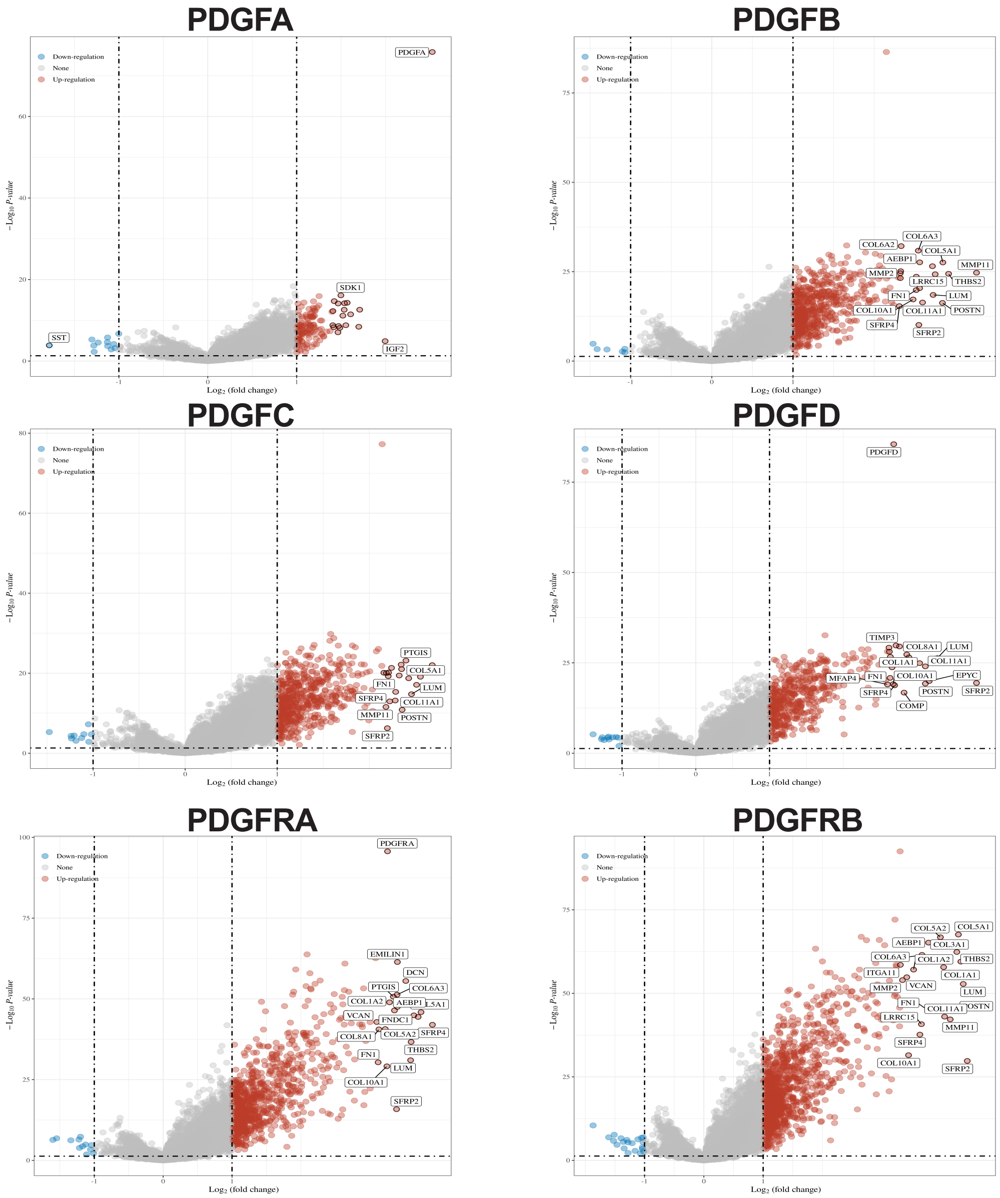

Figure S2: The volcano plot of DEGs between the high-expression group and the low-expression group of PDGF family members in ovarian cancer.
